# Clinical Course and Outcome of Prenatally Detected 22q11.2 Deletion Syndrome—A Retrospective Analysis

**DOI:** 10.3390/diagnostics13132244

**Published:** 2023-07-01

**Authors:** Chiara Paternostro, Stephanie Springer, Gregor Kasprian, Gülen Yerlikaya-Schatten, Theresa Reischer

**Affiliations:** 1 Division of Obstetrics and Feto-Maternal Medicine, Department of Obstetrics and Gynaecology, Medical University of Vienna, 1090 Vienna, Austria; chiara.paternostro@meduniwien.ac.at (C.P.); stephanie.springer@meduniwien.ac.at (S.S.); theresa.reischer@meduniwien.ac.at (T.R.); 2 Division of Neuroradiology and Musculoskeletal Radiology, Department of Radiology, Medical University of Vienna, 1090 Vienna, Austria; gregor.kasprian@meduniwien.ac.at

**Keywords:** microdeletion syndrome, 22q11.2 deletion syndrome, DiGeorge syndrome, congenital heart defect, prenatal MRI, postmortem examination

## Abstract

The 22q11.2 deletion syndrome (22q11.2 DS) is known as the most common microdeletion syndrome. Due to its variable clinical phenotype, prenatal diagnosis can be challenging. The aim of this retrospective study was to evaluate the clinical course and pregnancy outcome of cases with prenatally diagnosed 22q11.2 deletion syndrome (DS) as well as to evaluate the role of prenatal magnetic resonance imaging (MRI) and postmortem examination. In total, 21 cases who underwent prenatal ultrasound examination and pregnancy care at the Department of Obstetrics and Gynecology at the Medical University of Vienna between 2012 and 2022 were included. The majority of the cases were genetically diagnosed using fluorescent in situ hybridization (FISH). The median gestational age (GA) at genetic diagnosis was 23.0 weeks (IQR 21.4–24.8 weeks). CHDs were detected in all fetuses and the most common extracardiac manifestation was thymus hypo/aplasia followed by genitourinary anomalies. Prenatal magnetic resonance imaging (MRI) revealed additional diagnostic information in three of ten cases. Overall, 14 patients opted for drug-induced TOP, of which 9 cases had a feticide prior to the induction of labor. The majority of craniofacial malformations were only detected by autopsy. In conclusion, the majority of cases prenatally diagnosed with 22q11.2 DS had an absent or hypoplastic thymus noted antenatally in addition to the detected CHD, and almost half of the cases had another extracardiac malformation of predominantly genitourinary origin. Furthermore, prenatal MRIs confirmed previously detected malformations, but only provided additional diagnostic information in three out of ten cases, whereas postmortem examination diagnosed most of the craniofacial anomalies and should always be conducted, serving as an important quality indicator for prenatal imaging.

## 1. Introduction

The 22q11.2 deletion syndrome (22q11.2 DS) is known as the most common microdeletion syndrome with a prevalence of 1:3000–1:6000 in live births [1,2,3]. It is associated with multi-organ anomalies like congenital heart defects (CHDs), thymic anomalies leading to immunodeficiency, abnormal facies, cleft palate, hypoparathyroidism, and cerebral anomalies [4,5,6]. Due to its variable clinical phenotype, prenatal diagnosis can be challenging. Previous studies showed that 22q11.2 DS was detected in 1 out of 19 pregnancies that underwent invasive genetic testing for detected cardiac abnormalities, and that it is the second most common cause of CHD in general [7,8,9]. About 60–80% of affected individuals suffer from CHDs of a conotruncal origin like tetralogy of Fallot (TOF), truncus arteriosus, or interrupted aortic arch (IAA) [10,11]. Furthermore, in the case of conotruncal CHD, the size of the thymus should always be examined in detail, as a concomitant manifestation is known to be a major finding indicating 22q11.2 DS [12,13]. Fetal malformations associated with 22q11.2 are detected mostly in the second trimester and early prenatal diagnosis can help to enable fully informed decision making regarding further pregnancy management [14]. Approximately 85% of 22q11.2 DS occur as de novo deletions most commonly involving 2.5 to 3 mega bases (Mb), whereas smaller nested deletions are found considerably less frequently [8]. Diagnostic confirmation in the case of suspected 22q11.2 can be obtained through specific genetic testing such as fluorescent in situ hybridization (FISH), multiplex ligation-dependent probe amplification (MLPA), and/or chromosomal microarray (CMA) [15].

Prenatal magnetic resonance imaging (MRI) has become an important diagnostic tool in prenatal imaging, especially in the detection of central nervous system (CNS) anomalies, and can provide important information influencing further patient counseling and clinical management [16,17]. Although diagnostic features and challenges of prenatally detected 22q11.2 DS by ultrasound (US) are well described, studies investigating the diagnostic utility of fetal MRI with this specific congenital condition are lacking [18].

The aim of this study was to investigate the clinical course and pregnancy outcome of all fetuses with prenatally diagnosed 22q11.2 DS. Furthermore, we sought to evaluate the role of prenatal MRI in the assessment of fetal anomalies and compare prenatal imaging findings with postmortem examinations in this specific condition.

## 2. Materials and Methods

In this retrospective single-center cohort study, 21 cases with prenatally diagnosed 22q11.2 DS were included. The inclusion criteria were pregnant women with prenatally diagnosed and genetically confirmed fetal 22q11.2 DS, who underwent prenatal ultrasound examination and pregnancy care at the Department of Obstetrics and Gynecology at the Medical University of Vienna between January 2012 and December 2022. General medical history, fetal, and maternal characteristics were extracted from our perinatal database retrospectively. Chorionic villus sampling (CVS) for karyotyping and CMA were offered in the first trimester to all cases with structural abnormalities or a nuchal translucency above 3.5 mm. In the second trimester, amniocentesis (AC) or a placental biopsy (PB) for karyotyping and CMA was offered to all cases with a structural fetal malformation. Independent of the indication of whether to proceed with invasive testing, FISH analysis for trisomy 21, 13, and 18 was performed as a first line test, whereas a specific FISH analysis for 22q11.2 DS was conducted additionally only in the case of a CHD. MLPA was rarely used, i.e., only in order to confirm a previous detection of 22q11.2 or when a specific FISH analysis for 22q11.2 DS was not available.

If available, the deletion size was reported in mega bases (Mb), and a deletion size between 2.5 and 3.0 Mb was referred to as a “typical deletion”. A non-invasive prenatal screening test (NIPT) for 22q11.2 DS was not performed in any of the cases. In the case of the genetic (co-)testing of the parents, the blood samples were taken on the same day of invasive testing. A detailed scan conducted by a fetal medicine specialist was performed at least once in every case. A prenatal MRI was offered to all of the women. In the case of termination of pregnancy (TOP), labor was induced with mifepristone and misoprostol. For TOP after 22 + 0 weeks of gestation, a feticide was offered, whereas after 23 + 0 weeks, a feticide prior to TOP was performed to avoid live births. Feticides were performed mainly via an intracardiac injection of either potassium chloride or lidocaine; in one case, due to fetal position, the injection was performed through the umbilical cord. Every TOP for 22q11.2 DS was discussed by an internal clinical committee and an autopsy was performed in all cases. The pregnancy outcome was defined as live birth or TOP with or without feticide. The number of cases of spontaneous abortion or intrauterine fetal death were reported. Fetal anomalies were classified into the following categories: “congenital heart defect”, “thymic hypo/aplasia”, “skeletal dysplasia”, “genitourinary”, “cerebral”, “craniofacial”, or “abdominal”. Each case was assigned a sequential number for identification and facile citation in the text.

The nominal variables are reported as numbers and frequencies, and continuous variables are reported as medians and interquartile ranges (IQRs). Statistical analyses were performed with SPSS v26 (released 2019, IBM Corp., Armonk, NY, USA) using the Mann–Whitney U test or the Fisher’s exact test, where appropriate. A two-sided *p* value < 0.05 was considered significant.

## 3. Results

In this retrospective analysis of a single tertiary prenatal center with an average of 2650 live births and 41 stillbirths per year, we evaluated 21 cases with prenatally diagnosed 22q11.2 DS over a period of 11 years.

### 3.1. Clinical Course

The median maternal age was 33 years (IQR 31–35 years). Two cases of assisted reproductive technology (ART) were reported and most women were primiparous. For detailed maternal characteristics see Table 1.

The median GA at the detection of abnormal findings by US was 21.9 weeks, ranging from 11.0 to 24.4 weeks, and invasive genetic testing was performed due to abnormal ultrasound findings in all cases. In 19 cases, invasive testing was performed due to the detection of a CHD, and, in two cases, due to an abnormal FTS. The median GA at genetic diagnosis was 23.0 weeks (IQR 21.4–24.8 weeks, Table 2).

Detailed information about the distribution of cardiac and extracardiac malformations can be found in Table 3, Table 4 and Table 5. 

First trimester screening (FTS) was performed in 11 women. In two patients, FTS showed an abnormal finding. In one woman, FTS detected bilateral hydronephrosis, which was confirmed in the follow-up scans (Table 5; case 17), and in another woman a cystic hygroma and an increased nuchal translucency with 3.4 mm was described (Table 5, case 18).

**Table 5:** Detailed prenatal and postmortem examination findings of fetuses with 22q11.2 deletion syndrome in TOP without feticide.

Case Number	GA at Live Birth/TOP, Weeks	Genetic Test for 22q11.2 DS	Deletion Size, Mega Bases	Inheritance	CHD	Thymus Hypo/Aplasia	Genitourinary Anomalies	Skeletal Dysplasia	Cranifacial Anomalies	Cerebral Anomalies	Abdominal Anomalies
TOP without feticide											
17	20.3	FISH, CMA	2.53	maternal 22q11.2 DS	RAA	yes	Bilateral hydronephrosis	-	-	-	-
18	20.1	FISH	-	unknown	TOF	yes	-	-	Hypertelorism	-	-
19	22.1	FISH	-	unknown	TOF	yes	-	-	Hypertelorism, low-set ears, retrognathia	-	-
20	21.9	FISH, CMA	2.2	de novo	Truncus arteriosus	yes	-	-	-	-	-
21	16.4	FISH	-	de novo	Truncus arteriosus	yes	Bilateral renal agenesis	Malposition and contractures of upper and lower extremities	-	-	-

Abbreviations used: GA, gestational age; TOP, termination of pregnancy; FISH, fluorescent in situ hybridization; MLPA, multiplex ligation-dependent probe amplification; CMA, chromosomal microarray; CHD, congenital heart defect; TOF, Tetralogy of Fallot, RAA, right aortic arch; IAA, interrupted aortic arch; VSD, ventricular septal defect; DORV, double-outlet right ventricle; TGA, transposition of the great arteries; PA, pulmonary artery.

In ten cases, the fetuses were female. CHDs were detected via ultrasound in all of the fetuses, with Tetralogy of Fallot (TOF) observed most often (*n* = 8/21; Table 6). The most common extracardiac malformation was thymus hypo/aplasia, which was diagnosed in 19 fetuses via ultrasound, but could not be evaluated prenatally due to fetal position or GA in two cases, followed by genitourinary anomalies detected in seven cases with uni- or bilateral hydronephrosis being diagnosed most often. The majority of genitourinary anomalies (*n* = 5/6) were diagnosed via US, whereas one case with cryptorchidism was detected via autopsy only. In two cases, cerebral malformations were observed, of which neurosonography diagnosed one case with a mega cisterna magna.

Furthermore, a polyhydramnios was found in eight cases, and, in one case, a single uterine artery (SUA) was detected. Isolated ultrasound findings of the heart occurred in two cases (Table 5, case 18 and 21), whereas extracardiac anomalies were observed only as associated multiple anomalies. Twelve fetuses were prenatally diagnosed with CHD and thymus hypo/aplasia without evidence of other extracardiac manifestations on the US, of which eight cases were found to have an additional anomaly detected by autopsy. 

**Table 6:** Distribution of congenital heart defects diagnosed via ultrasound.

CHD	All Cases (*n* = 21)	Live Births (*n* = 7)	TOP (*n* = 14)
TOF	8 (38.1%)	2 (28.6%)	6 (42.9%)
RAA	4 (19.0%)	2 (28.6%)	2 (14.3%)
IAA	3 (14.3%)	1 (14.3%)	2 (14.3%)
Truncus arteriosus	3 (14.3%)	0 (0%)	3 (21.4%)
VSD, aortic arch hypoplasia	2 (9.5%)	1 (14.3%)	1 (7.1%)
DORV, TGA, PA hypoplasia	1 (4.8%)	1 (14.3%)	0 (0%)

Data are presented as numbers (frequencies); Abbreviations used: GA, gestational age; CHD, congenital heart defect; TOF, Tetralogy of Fallot, RAA, right aortic arch; IAA, interrupted aortic arch; VSD, ventricular septal defect; DORV, double-outlet right ventricle; TGA, transposition of the great arteries; PA, pulmonary artery.

### 3.2. Fetal Anomalies Diagnosed via MRI

A prenatal MRI was performed in ten cases, of which three additionally revealed fetal anomalies not detected by previous US examinations. Regarding cerebral malformations, the MRI revealed a polymicrogyria and specified the US finding of a mega cisterna magna by suspecting an arachnoidal cyst. In one case, a prenatal MRI discovered a hepatosplenomegaly with a concomitant liver cyst and a cleft palate in another one. Thymus hypo/aplasia cases previously observed during an US were confirmed by an MRI in all fetuses. In the two cases in which the thymus could not be assessed via US, an MRI was not performed. Skeletal dysplasia, genitourinary malformations, as well as the presence of a polyhydramnios observed by US were also confirmed by MRI in all cases.

### 3.3. Genetic Testing

The median GA at invasive genetic testing, irrespective of the invasive technique performed, was 22.0 (21.0–23.0 weeks, Table 2) with 22.1 weeks for AC, ranging from 21.4 to 23.2 weeks, and 21.1 weeks for CVS/PB, ranging from 15.0 to 23.0 weeks, respectively. FISH for trisomy 21,18, and 13 revealed a negative result in all of the included cases. The genetic diagnosis was primarily confirmed through FISH analysis for 22q11.2 DS in twelve cases, CMA in nine cases, and MLPA in one patient. One deletion detected via FISH was confirmed through additional MLPA and CMA analyses (Table 3, case 5). Cases diagnosed via CMA were confirmed either using FISH (*n* = 7) or MLPA (*n* = 2). The size of the deletion was only reported when a CMA analysis was performed, i.e., in nine cases, ranging from 1.4 to 9.6 Mb. CMA detected a typical deletion in one case, nested deletions smaller than 2.5 Mb in six, and deletions larger than 3.0 Mb in two cases. Genetic testing of the parents was conducted in 15 cases. In two fetuses, the deletions were maternally inherited; in one case, a maternal balanced translocation of chromosome 8 and 22 led to a 9.6 Mb deletion of chromosome 22 in the fetus (Table 3, case 4), and, in the other case, the mother was diagnosed with 22q11.2 DS and inherited a 2.53 Mb deletion which she passed on to her fetus (Table 5, case 17). The mother herself was known to have a RAA and a concomitant unilateral hydronephrosis, although a definite genetic diagnosis was not made until genetic co-testing was performed during pregnancy.

### 3.4. Pregnancy Outcome and Postpartum Management

Overall, 14 patients opted for TOP, of which 9 cases had a feticide prior to the induction of labor. The median GA at TOP without feticide was 21.1 weeks (IQR 19.2–22.5) and with feticide it was 24.3 weeks (IQR 23.6–26.0; Table 2). The median fetal weight of fetuses in the case of TOP with feticide was 659 g (IQR 598–846 g) and 398 g (IQR 242–493 g) in cases without. The time between diagnosis and TOP was 1.3 weeks (IQR 1.0–2.1 weeks) in the case of TOP with feticide, and 1.1 weeks (IQR 0.9–2.6 weeks) in those without feticide. The GA at diagnosis was significantly lower in cases with TOP than in women opting for a continuation of pregnancy (21.8 weeks (IQR 19.8–23.3 weeks) vs. 25.3 weeks (IQR 23.1–26.9 weeks); *p*-value = 0.001)

Seven women opted for a continuation of pregnancy. Two cases underwent an amnioreduction due to severe polyhydramnios; in one woman (Table 3, case 1), genetic testing was performed during amnioreduction during week 30.0, in the other case (Table 3, case 4), an additional amnioreduction was performed during week 30.4 after a previous AC for genetic testing during week 24.4. In both cases, the polyhydramnios was of unknown origin. Women having live births delivered at a median GA of 38.0 weeks (IQR 37.3–29.6 weeks). In total, four women delivered by c-section. Two of those women had a primary c-section (one case due to a history of two previous c-sections and the other one upon the mother’s request) and the other two had a secondary c-section (both due to arrested labor). All of the neonates were transferred to the neonatal inpatient department directly after birth, and six out of seven underwent heart surgery within twelve months postpartum. During the analyzed postnatal follow-up period of 12 months, all seven cases survived.

### 3.5. Fetal Anomalies Diagnosed by Autopsy

A postmortem examination performed by a pathologist was conducted in all 14 cases after TOP. In 13 cases, the autopsy revealed fetal anomalies, which were not previously detected via ultrasound or MRI, and which were most commonly of craniofacial origin. Six cases with dysmorphic facies were discovered only by autopsy, with hypertelorism found in five, low-set ears in four, and micrognathia in four cases. In one case, in which the US detected retrognathia, the autopsy additionally revealed a hypertelorism and low-set ears (Table 5, case 19). One fetus with a cleft palate, diagnosed using a prenatal MRI, was found to have hypertelorism, low-set ears, and micrognathia following postmortem examination (Table 4, case 13). In addition, autopsy revealed two cases of thymus hypo/aplasia previously not detected by the US (Table 5, case 19 and 21), skeletal dysplasia with malposition of the extremities and/or contractures in another two fetuses (Table 4, case 11 and Table 5, case 21), and cryptorchidism in one case (Table 4, case 16).

## 4. Discussion

In this single center retrospective study, we evaluated 21 pregnant women with prenatally diagnosed fetuses with 22q11.2 DS over a time of 11 years, of which 66.7% opted for a TOP. Most of the fetal anomalies were detected via US, prenatal MRI provided additional diagnostic findings in three out of ten cases, and the majority of craniofacial anomalies were detected via autopsy.

One of the eleven performed FTSs showed an increased NT of 3.4 mm, which is comparable with other studies, indicating an increased NT frequency ranging from 5 to 9% [8]. The majority of fetal anomalies were detected using US during the second or third trimester at a median GA of 21.9 weeks, which is also similar to previous observations in other studies [14,19]. CHDs were found in all of the 21 fetuses, with conotruncal defects observed most frequently. Since cardiovascular malformations are the leading indications for invasive testing in 22q11.2 DS with a prenatal detection rate of 62–95%, affected fetuses without cardiovascular defects may only be diagnosed after birth or in early childhood, explaining the lower rate of cardiovascular malformations in postnatal studies of 64% [8,20]. In our cohort, we found a relatively low rate of isolated CHD, i.e., 9.5%, probably due to the high rate of detected thymic anomalies. In the majority of the cases included in this analysis (*n* = 12), thymic hypo/aplasia was the only extracardiac abnormality diagnosed prenatally, whereas in seven cases another extracardiac malformation was detected in addition to cardiac and thymic defects. In previous reports, the rate of isolated CHD was up to 62% of the cases, while isolated extracardiac findings were detected significantly less often [14,21].

Non-invasive prenatal testing (NIPT) as a screening for microdeletion syndromes has gained importance in recent years, although it is not yet offered as a routine test due to having a low sensitivity of 75% and low positive predictive value (PPV) of 23.7% for 22q11.2 in particular [1]. Nevertheless, the rate of prenatally detected 22q11.2 DS without abnormal US findings is likely to increase substantially during the coming years, as the use of NIPT as well as its diagnostic accuracy for microdeletion syndromes are expected to change [22,23].

Thymic hypo- or aplasia was also detected in every fetus, and almost all of the cases were diagnosed using US (90.5%). Compared to other articles, this detection rate is considerably higher than the previously described frequency, ranging from 3 to 68% [14,19,21,24].

A fetal MRI was performed in 47.6% of cases, and revealed additional diagnostic information in three cases, detecting a cleft palate, polymicrogyria, and a liver cyst with concomitant hepatosplenomegaly. Cardiovascular anomalies were usually not evaluated in detail since the diagnostic sensitivity of an MRI in congenital heart disease is known to be lower than on fetal echocardiography [25]. The other fetal anomalies found via US were all specifically evaluated and confirmed using an MRI. Thus, in our data, MRI seems to be an important supplementary diagnostic tool to confirm fetal anomalies previously found on US, but it was of limited value in regard to providing an additional diagnostic benefit. Nevertheless, prenatal MRI can facilitate the general diagnostic assessment of fetuses with multiple and complex anomalies, as often seen in 22q11.2 DS, and therefore may lead to more precise prenatal counseling. Furthermore, various studies have reported a higher diagnostic accuracy of fetal brain MRI compared with neurosonography, leading to a change in clinical management due to the MRI findings in up to 30.2% cases [16,26]. Since central nervous system anomalies are described to occur in 38% of individuals with 22q11.2 DS, a prenatal MRI should be offered to all affected families, if available, to avoid overlooking CNS anomalies, which will, most probably, have a great impact on pregnancy management decisions and prognosis counseling [21].

CMA is currently used as the gold-standard method to detect microdeletion syndromes due to having a high diagnostic accuracy locating also atypical or smaller deletions, whereas FISH only detects typical sized deletions [27,28]. On the other hand, FISH analysis is known to be a much faster method than CMA, increasing the chances of prompt prenatal genetic confirmation within a few days. Most of the 22q11.2 DS in our cohort were primarily confirmed via the FISH technique, whereas CMA mainly detected smaller deletions. Although CMA has been offered routinely in selected cases since 2011 in our perinatal center, most of the deletions detected using FISH were not re-confirmed via an additional CMA, leading to less cases with specified deletion sizes. In accordance with the previous reports, most of the patients showed a typical deletion (including eight deletions detected by FISH and one by CMA), and 13.3% of 22q11.2 DS in our cohort were maternally inherited [14,29].

Fourteen women (66.7%) decided to terminate their pregnancy, with more than half (*n* = 9) of the cases undergoing a previous feticide. This rate is comparable with previous reports, indicating a TOP frequency between 69–79% with a lack of additional information on feticide rates [14,19]. Most of our cases underwent invasive testing at 21 or more weeks (*n* = 18). Considering a diagnostic delay of 7–14 days until the final genetic results were available, most decisions to terminate or continue the pregnancy were made at the borderline of viability. The diagnosis of 22q11.2 DS by genetic confirmation was made significantly earlier in women who chose TOP compared to women who continued their pregnancy, demonstrating an association between an earlier diagnosis and higher rates of TOP, which was also observed previously in the literature [14].

The postmortem investigation revealed a considerable amount of additional information in 92.9% of cases. The majority of craniofacial anomalies were only diagnosed via autopsy, which conforms with the findings of Besseau-Ayasse J. et al., who found a similar rate of 72.9% of additional diagnostic information related to facial defects [14]. A systematic review published by Rossi C. and Prefumo F. analyzed over 3500 fetuses undergoing autopsy and found that 22.5% of the post mortem examinations revealed additional information on fetal anomalies formerly missed in the US, which supports the recommendation that autopsy should be performed routinely, especially after TOP [30,31,32,33]. In our cohort, an autopsy rate of 100% was achieved, as postmortem examination after TOP is required by law in Austria.

One of the main limitations of this study is its retrospective character, with data collected using record review and a lack of prospective information. Another limitation is the heterogeneous genetic workup due to the availability of genetic analysis at different timepoints leading to a lack of information on specific deletion sizes. To our knowledge, this is the first study evaluating the impact of fetal MRI on the prenatal diagnosis of 22q11.2 DS, which we consider as the major strength of our analysis, as well as the high rate of postmortem examinations, which allowed us to assess the correlation between prenatal imaging and postmortem autopsy.

## 5. Conclusions

In conclusion, we demonstrated that 9.5% of fetuses with 22q11.2 DS were prenatally found to have isolated CHD, whereas the remaining cases had extracardiac malformations in addition to the primary cardiac defect, with 57.1% having thymic anomalies only, and 42.9% having thymic and other extracardiac malformations of predominantly genitourinary origin. It is noteworthy that in our cohort, extracardiac anomalies never occurred in isolation. Furthermore, prenatal MRI confirmed previously detected malformations in all fetuses, but only provided additional diagnostic information in three out of ten cases, whereas postmortem examination diagnosed most of the craniofacial anomalies and should always be conducted, serving as an important quality indicator for prenatal imaging.

## Figures and Tables

**Table 1:** Basic maternal characteristics.

	*n* = 21
Maternal age, years ^a^	33 (31–35)
Maternal BMI ^a^	23 (21–26)
Primiparous ^b^	14 (66.7%)
Prenatal MRI ^b^	10 (47.6%)
CVS/PB ^b^	6 (28.6%)
AC ^b^	15 (71.4%)
ART ^b^	2 (9.5%)

Data are presented as ^a^ median (interquartile range) or ^b^ numbers (frequencies); abbreviations used: BMI, body mass index; MRI, magnetic resonance imaging; CVS, chorionic villus sampling; PB, placental biopsy; AC, amniocentesis; ART, artificial reproductive technology.

**Table 2:** Fetal characteristics and anomalies detected prenatally via ultrasound.

	All Cases (*n* = 21)	Live Births (*n* = 7)	TOP (*n* = 14)
GA at invasive testing, weeks ^a^	22.0 (19.7–22.9)	23.3 (22.0–25.0)	21.4 (16.6–22.1)
GA at diagnosis, weeks ^a^	23.0 (21.4–24.8)	25.3 (23.1–26.9)	21.8 (19.8–23.3)
CHD ^b^	21 (100%)	7 (100%)	14 (100%)
thymus hypo/aplasia ^b^	19 (90.4%)	7 (100%)	12 (85.7%)
genitourinary anomaly ^b^	5 (23.8%)	3 (42.9%)	2 (14.3%)
cerebral anomaly ^b^	2 (9.5%)	1 (14.3%)	1 (7.1%)
skeletal dysplasia ^b^	1 (4.8%)	0 (0%)	1 (7.1%)
craniofacial anomaly ^b^	1 (4.8%)	0 (0%)	1 (7.1%)
abdominal anomaly ^b^	1 (4.8%)	1 (14.3%)	0 (0%)
polyhydramnios ^b^	8 (38.1%)	5 (71.4%)	3 (21.4%)

Data are presented as ^a^ median (interquartile range) or ^b^ numbers (frequencies); abbreviations used: GA, gestational age; CHD, congenital heart defect.

**Table 3:** Detailed pre- and postnatal examination findings of fetuses with 22q11.2 deletion syndrome in live births.

Case Number	GA at Live Birth/TOP, Weeks	Genetic Test for 22q11.2 DS	Deletion Size, Mega Bases	Inheritance	CHD	Thymuys Hypo/Aplasia	Genitourinary Anomalies	Skeletal Dysplasia	Cranifacial Anomalies	Cerebral Anomalies	Abdominal Anomalies
Live birth											
1	39.6	FISH, CMA	2.16	de novo	TOF	yes	Unilateral polycystic kidney	-	-	-	-
2	38.4	FISH, CMA	2.25	de novo	TOF	yes	Unilateral hydronephrosis	-	-	Polymicrogyria	-
3	37.9	MLPA, CMA	3.2	de novo	VSD, aortic arch hypoplasia	yes	-	-	-	-	-
4	36.4	MLPA, CMA	9.6	maternal translocation 8/22	DORV, TGA, PA hypoplasia	yes	-	-	-	-	-
5	38.0	FISH, MLPA, CMA	-	de novo	IAA	yes	-	-	-	-	-
6	40.9	FISH	-	unknown	IAA	yes	Unilateral hydronephrosis	-	-	-	-
7	37.2	MLPA	-	de novo	RAA	yes	-	-	-	-	-

Abbreviations used: GA, gestational age; TOP, termination of pregnancy; FISH, fluorescent in situ hybridization; MLPA, multiplex ligation-dependent probe amplification; CMA, chromosomal microarray; CHD, congenital heart defect; TOF, Tetralogy of Fallot, RAA, right aortic arch; IAA, interrupted aortic arch; VSD, ventricular septal defect; DORV, double-outlet right ventricle; TGA, transposition of the great arteries; PA, pulmonary artery.

**Table 4:** Detailed prenatal and postmortem examination findings of fetuses with 22q11.2 deletion syndrome in TOP with feticide.

Case Number	GA at Live Birth/TOP, Weeks	Genetic Test for 22q11.2 DS	Deletion Size, Mega Bases	Inheritance	CHD	Thymuys Hypo/Aplasia	Genitourinary Anomalies	Skeletal Dysplasia	Cranifacial Anomalies	Cerebral Anomalies	Abdominal Anomalies
TOP with feticide											
8	24.9	FISH, CMA	2.18	de novo	TOF	yes	-	-	-	-	-
9	24.6	FISH, CMA	2.2	de novo	RAA	yes	-	-	Hypertelorism	-	-
10	26.0	FISH, CMA	1.4	de novo	TOF	yes	-	-	Hypertelorism, low-set ears	-	-
11	23.6	FISH	-	de novo	VSD, aortic arch hypoplasia	yes	-	Malposition of upper extremities	Low-set ears, micrognathia	Mega cisterna magna	-
12	26.0	FISH	-	unknown	IAA	yes	-	-	Hypertelorism, low-set ears, micrognathia	-	Liver cyst, hepatosplenomegaly
13	26.4	FISH	-	de novo	Truncus arteriosus	yes	-	-	Hypertelorism, low-set ears, micrognathia, mild cleft palate	-	-
14	22.3	FISH	-	unknown	RAA	yes	-	Talipes	-	-	-
15	23.6	FISH	-	unknown	TOF	yes	-	-	Hypertelorism, low-set ears, micrognathia	-	-
16	24.0	FISH		de novo	TOF	yes	Cryptorchidism	-	-		

Abbreviations used: GA, gestational age; TOP, termination of pregnancy; FISH, fluorescent in situ hybridization; MLPA, multiplex ligation-dependent probe amplification; CMA, chromosomal microarray; CHD, congenital heart defect; TOF, Tetralogy of Fallot, RAA, right aortic arch; IAA, interrupted aortic arch; VSD, ventricular septal defect; DORV, double-outlet right ventricle; TGA, transposition of the great arteries; PA, pulmonary artery.

## Data Availability

The data presented in this study are available on request from the corresponding authors.
